# Electrical Stimulation Promotes Wound Healing by Enhancing Dermal Fibroblast Activity and Promoting Myofibroblast Transdifferentiation

**DOI:** 10.1371/journal.pone.0071660

**Published:** 2013-08-19

**Authors:** Mahmoud Rouabhia, Hyunjin Park, Shiyun Meng, Habib Derbali, Ze Zhang

**Affiliations:** 1 Faculty of Dentistry, Research Group on Oral Ecology, Laval University, Quebec City, Quebec, Canada; 2 Faculty of Medicine, Department of Surgery, Laval University, Saint-François d’Assise Hospital Research Center, CHU, Quebec City, Quebec, Canada; 3 College of Environment and Biotechnology, Chongqing Technology and Business University, Chongqing, China; University of Illinois at Chicago, United States of America

## Abstract

Electrical stimulation (ES) has long been used as an alternative clinical treatment and an effective approach to modulate cellular behaviours. In this work we investigated the effects of ES on human skin fibroblast activity, myofibroblast transdifferentiation and the consequence on wound healing. Normal human fibroblasts were seeded on heparin-bioactivated PPy/PLLA conductive membranes, cultured for 24 h, and then exposed to ES of 50 or 200 mV/mm for 2, 4, or 6 h. Following ES, the cells were either subjected to various analyses or re-seeded to investigate their healing capacity. Our findings show that ES had no cytotoxic effect on the fibroblasts, as demonstrated by the similar LDH activity levels in the ES-exposed and non-exposed cultures, and by the comparable cell viability under both conditions. Furthermore, the number of viable fibroblasts was higher following exposure to 6 h of ES than in the non-exposed culture. This enhanced cell growth was likely due to the ES up-regulated secretion of FGF-1 and FGF-2. In an *in vitro* scratch-wound assay where cell monolayer was used as a healing model, the electrically stimulated dermal fibroblasts migrated faster following exposure to ES and recorded a high contractile behaviour toward the collagen gel matrix. This enhanced contraction was supported by the high level of α-smooth muscle actin expressed by the fibroblasts following exposure to ES, indicating the characteristics of myofibroblasts. Remarkably, the modulation of fibroblast growth continued long after ES. In conclusion, this work demonstrates for the first time that exposure to ES promoted skin fibroblast growth and migration, increased growth factor secretion, and promoted fibroblast to myofibroblast transdifferentiation, thus promoting wound healing.

## Introduction

It is well known that the endogenous electrical field (EF) in the human body plays several critical physiological roles, including the electrical activation of the nervous system and the muscles. The human body also generates transepithelial electrical potential (TEP) ranging between 10 and 60 mV in various locations [1], [2]. This TEP is active in wound healing by promoting cell migration from the wound edges [3]. Injured epidermis is thus characterised by a TEP short circuit that gives rise to a measurable DC current efflux between 1 and 10 µA/cm^2^ and an estimated current density up to 300 µA/cm^2^ near the edge of the wound [3]–[5]. This wound current corresponds to a relatively steady local EF between 40 and 200 mV/mm. The EF persists until complete wound re-epithelialisation is achieved [4], [6]–[8].

The beneficial effect of this endogenous EF is to guide cell migration and nerve sprouting directly toward the wound edge; however, this healing process is compromised if the EF is inhibited [8]–[10]. It has also been shown that rapid uncontrolled cell proliferation causes significant changes in cell surface charge [11], thus tumours become polarised relative to quiescent surrounding regions. In breast cancer, for example, potential difference between the proliferating and non-proliferating regions was suggested as a diagnostic criterion because the regions would possibly correlate with neoplasm malignancy [12]. EF also induces angiogenic responses in endothelial cells by promoting cell elongation and directional migration. The effect of EF on endothelial cells may be mediated by vascular endothelial growth factor (VEGF)-receptor activation [13]. During the skin wound healing process, fibroblasts, epithelial cells and endothelial cells are actively involved in extra cellular matrix production (ECM) [14], the re-epithelialisation [3], [15] and angiogenesis [16]. Indeed, following an in vitro wound scratch assay, the injured cell monolayer responds to the disruption of cell-cell contacts through different mechanisms including cell growth and migration [17], [18], and an increased level of growth factors at the wound. These processes reflect the behavior of individual cells as well as the properties of the cell sheet as a surrogate tissue.

Because EF affects both epithelial and endothelial cells, and as these cells interact intimately with fibroblasts, we hypothesised that EF would have a modulating effect on fibroblasts and their role in the wound healing process. Weak DC EFs reportedly led to fibroblasts aligned perpendicularly to the EF lines [19], [20]. Primary fibroblasts also showed an increased migration rate following exposure to EF for 2 h [19]. Other studies reported that injury currents near wounds could last longer than 5 h, suggesting its role in directing cell migration [19]. Compared to other cells, fibroblasts require a longer exposure time (over 2 h) to low strength EF. Thus activating fibroblasts within a shorter period of time (less than 2 h) requires a field strength greater than 100 mV/mm. This particular EF exists *in vivo* [19].

In normal human tissue, fibroblasts contribute to tissue homeostasis by regulating the turnover of extracellular matrix (ECM). When the tissue is injured, fibroblasts proliferate and growth factors increase. For example, the induction of fibroblast growth factor FGF-1 and FGF-2 was shown to occur earlier in the wounds of diabetic animals than in those of non-diabetic animals, and the expression of both FGF-1 mRNA and FGF-2 mRNA returned to basal levels within 3 days after injury [21]. Some of the fibroblasts in the injured region may differentiate into a highly contractile phenotype, i.e., myofibroblasts, which also produce abundant ECM proteins.

Despite the fact that the mechanisms of wound healing are not completely understood, it has become evident that both fibroblasts and myofibroblasts play critical roles in wound healing. Specifically, the traction force of fibroblasts and the coordinated contraction of myofibroblasts are believed to be responsible for wound contraction and closure [22]. Myofibroblasts are a cell type phenotypically between fibroblasts and smooth muscle cells because they express alpha-smooth muscle actin (α-SMA), an early differentiation marker of smooth muscle cells [23]. Myofibroblasts are found in the early phase of granulation tissue formation [24], become most abundant during the proliferation phase of wound healing, and gradually disappear during the final stages of healing, presumably through an apoptotic process. In skin wounds, myofibroblasts originate from the locally recruited fibroblasts in the dermis and subcutaneous tissue surrounding the wound, and contribute to wound healing [25].

Multiple strategies have been used to promote wound healing, including vacuum-assisted wound closure [26], shock-wave therapy [27], and local warming [28], to name a few. ES has been used as an adjunct to increase tissue blood flow and promote wound healing [29]. ES has been shown to promote cell growth and increase the healing rate of wounds [30], [31]. However, the specific effect of ES on fibroblasts as a key player in wound healing remains to be investigated. The aim of the present study was thus to evaluate the effect of ES on human skin fibroblast viability, growth factor secretion, migration, contractile activity and α-SMA expression.

## Materials and Methods

### 1. Human Skin Fibroblast Cultures

Normal human skin fibroblasts were obtained from ScienCell Research Laboratories (Carlsbad, CA, USA) and were cultured in Dulbecco’s modified Eagle’s medium (DME, Invitrogen Life Technologies, Burlington, ON, Canada) supplemented with fetal bovine serum (FBS) to a final concentration of 10% (Gibco, Burlington, ON, Canada). The medium was changed three times a week. When the cultures reached 90% confluence, the cells were detached from the flasks with a 0.05% trypsin-0.1% ethylenediaminetetraacetic acid (EDTA) solution, washed twice, and resuspended in FBS-supplemented DME medium. In each experiment, the fibroblasts were used between passages 4 and 5.

### 2. Preparation of the Heparin-bioactivated Conductive PPy/PLLA Membranes

To prepare the heparin (HE)-bioactivated polypyrrol (PPy) particles, freshly vacuum-distilled pyrrole monomers (98%, Aldrich Chemicals, Milwaukee, WI, USA) were added dropwise into a water-in-oil (chloroform) (3∶7) emulsion system containing 0.5 mg of HE (MW: 13,500–15,000; porcine sodium salt, Cat. 375095, EMD Biosciences, La Jolla, CA, USA) and Fenton’s reagent made by H_2_O_2_ and FeCl_3_ (Laboratoire Mat, Québec, QC, Canada) in nitrogen atmosphere. The emulsifier was 1%-dodecylbenzenesulfonic acid sodium salt (DBS; Sigma-Aldrich, St. Louis, MO, USA). Polymerisation was run at room temperature for 24 h under vigorous stirring. The PPy/HE particles were then precipitated with methanol and washed repeatedly with a mixture of H_2_O and methanol at a ratio of 1∶1 to remove the emulsifier and other impurities. The final PPy/HE particles were harvested after drying under vacuum at ambient temperature. To prepare the PPy/HE/PLLA membranes, the PPy/HE particles were added into the PLLA (η = 1.3 dL/g, Hycail B.V., Noordhorn, The Netherlands) solution in chloroform under stirring for 24 h to ensure homogeneity, followed by solution casting onto a polytetrafluoroethylene plate and final drying [32]. The thickness of the resulting membranes was approximately 0.5 mm. The final concentration of HE in the PPy/HE/PLLA membrane was approximately 0.9 mg/cm^2^. All of the membranes were kept in a desiccator until use.

### 3. Fibroblast Culture under Electrical Stimulation

Prior to cell seeding, the PPy/HE/PLLA membranes and the specially designed electric cell culture plates were sterilised with ethylene oxide gas at 37°C for 24 h according to standard industrial procedures. Thereafter, the PPy/HE/PLLA membranes were inserted into the culture device and pre-incubated with DME medium for 24 h to release any possible leachables from the membranes. To enable cell adhesion, skin fibroblasts (1×10^5^) were seeded in 3 ml of medium per each PPy/HE/PLLA membrane (ca. 4 cm^2^) and were incubated under 5% CO_2_ at 37°C for 24 h prior to ES. Following the renewal of the culture medium at 24 h, the PPy/HE/PLLA membranes were connected to a DC constant potential source through external electrodes. Two potential intensities were tested is this study, namely, 50 and 200 mV/mm. The cells were exposed to ES for 2, 4, or 6 h and were further cultured for 24 h prior to analysis. Sham control groups followed the same conditions except for exposure to ES. A minimum of four experiments was performed for each condition.

### 4. Cytotoxicity Assay

Cell injury was assessed by measuring lactate dehydrogenase (LDH) activity in the culture supernatant by means of an LDH cytotoxicity assay (Promega, Madison, WI, USA), as per the manufacturer’s protocol. Briefly, 50 µl of each supernatant were transferred to a 96-well flat-bottom plate, supplemented with 50 µl of reconstituted substrate mix, and then incubated in the dark at room temperature for 30 min. This assay is based on the conversion of L-lactate and NAD to pyruvate and NADH by the released LDH [33]. To stop the reaction, 50 µl of an acid solution was added to each well. The absorbance of each solution was read at 490 nm with an X-Mark microplate spectrophotometer (Bio-Rad, Mississauga, ON, Canada). A positive control (PC) for total LDH activity release was added to the experiment and was obtained by culturing cells in the presence of 1% Triton X-100. A negative control (NC) was obtained by culturing cells without exposure to ES. LDH activity release was calculated using the following formula: % of total LDH activity release = ((ES_absorbance_−NC_absorbance_)/(PC_absorbance_−NC_absorbance_))×100%.

### 5. ES Effect on Fibroblast Viability

At the end of each experiment, live cell numbers were determined. Briefly, fibroblasts were detached from the PPy/HE/PLLA membranes using 0.05% trypsin–0.01 EDTA solution. Following enzyme treatment, the cells were washed twice with 10% FBS-supplemented DME medium, and the resulting pellet was resuspended in 1 ml of DME medium and used to determine cell viability by trypan blue exclusion [34]. For this purpose, 100 µl of the cell suspension was mixed with the same volume of trypan blue solution and subsequently incubated on ice for 5 min, after which time the total number of cells in each sample and their viability were determined by trypan blue exclusion. Viable cells refer to cells that did not integrate the trypan blue. Results are reported as means ± SD of six assays.

### 6. Cell Migration/Monolayer Wound Repair Assay

*In vitro* wound repair assays were performed as previously described [35]. Briefly, 24 h after exposure to ES, the fibroblasts were detached from each conductive membrane, seeded (3×10^4^/well) in 6-well plates, and grown to confluence, after which time a scratch (wound) was created on each confluent monolayer using a 200 µl sterile pipette tip (PipetTipFinder, A division of Lab Procurement Services, LLC, Knoxville, TN, USA) perpendicular to the bottom of the dish. This generated a wound about 0.44 to 0.50 mm in width. The cultures were refreshed with new medium and were maintained under incubation. Digital photographs of each wound were taken (Coolpix 950; Nikon, Canada, Montréal, QC, Canada) at various time points following creation of the wound. Wound closure (cell migration) was investigated by measuring the distance between the opposite edges of the wound as the function of time using the NIH ImageJ public domain image processing program. Data (means ± SD, *n* = 5) are presented as the percentage of the initial wound (distance at time zero) using the following formula: ((distance at initial scratch – distance after an identified culture period) ÷ (distance at initial scratch)) × 100%. The ES-exposed and non-exposed cultures were compared, with the difference considered significant at p<0.05.

### 7. FGF-1 and FGF-2 Assay

For the cultures used to test FGF-1 and FGF-2 in supernatant, following ES the medium was replaced with a 1% FBS-supplemented DME medium and the cells were cultured for an additional 24 h. The supernatants were then collected to quantify the concentrations of FGF-1 and FGF-2 using the sandwich enzyme-linked immunosorbent assay (ELISA, R&D Systems, Minneapolis, MN, USA). The supernatants were collected in tubes containing 1 µl of a protease inhibitor cocktail for specific use with mammalian cells and tissue extracts (Sigma-Aldrich). Immediately thereafter, the supernatants were filtered through 0.22 µm filters and used to measure the growth factor levels. ELISA plates were read at 450 nm and were analysed by means of a Microplate Reader Model 680 (Bio-Rad, Philadelphia, PA, USA). The minimum detectable concentrations were under 6 pg/ml for FGF-1, and 0.07 pg/ml for FGF-2, as reported by the manufacturer. Each experiment was repeated four times and the means ± SD were calculated and presented.

### 8. Collagen Gel Contraction

The effect of ES on the capacity of human fibroblasts to contract collagen gel was determined using a reconstituted type I collagen assay system, as previously described [35]. Both the ES-exposed and non-exposed fibroblasts were detached from the conductive membranes and were used to measure collagen gel contraction. Briefly, fibroblasts (3×10^5^) were first mixed with rat tail collagen I (Cat. No. A 10483-01, Gibco-Invitrogen, Burlington, ON, Canada) and then poured onto 35 mm-diameter tissue culture plates. Following collagen-gel polymerisation, 3 ml of DME medium were added to each well and the resulting constructs were cultured in a 5% CO_2_ humid atmosphere at 37°C. The contraction of each collagen gel specimen was measured and photographed at various time points. This experiment was repeated four times.

### 9. Immunofluorescent Staining for α-SMA

Following ES and culture for 24 h, fibroblasts were detached from the PPy/HE/PLLA membranes, washed twice, and subsequently counted. They were then seeded on coverslips and grown to 60–70% confluence, after which time they were fixed with 4% paraformaldehyde and permeabilised with 0.3% Triton X-100. The immunofluorescent staining was performed by incubating cells with primary antibody against α-SMA (1∶100, clone 1A4, Sigma-Aldrich) for 60 min. The cells were washed three times with PBS and incubated thereafter with FITC-conjugated goat anti-mouse IgG and IgM antibody (1∶200, EMD Millipore Corporation, Billerica, MA, USA) for 45 min. Following this staining, the cells were washed with PBS and finally mounted with aqueous mounting medium. The stained cells were viewed under a fluorescence microscope and photographed.

### 10. Fibroblast Passage (Subculture) Following Exposure to ES

This experiment was designed to investigate the post-ES effect on fibroblast growth, and more specifically, the effect of ES on cell growth several days post-exposure. Following ES and 24 h of culture, fibroblasts were detached from the conductive membranes with trypsin, washed twice with culture medium, and subsequently counted. The cells were then placed in a 6-well plate at 3×10^4^ cells per well and cultured in a 5% CO_2_ humid atmosphere at 37°C for 24, 48, and 72 h, after which time the cells were detached and used to determine the number of viable cells by trypan blue exclusion assay, as described previously. Results are reported as means ± SD (n = 6). Viable cell numbers were also used to determine the rate of cell growth. The rate of cell growth at 48 h and 72 h was calculated by dividing the viable cell numbers at 48 h and 72 h with those at 24 h and 48 h, respectively. This was done for all conditions (no ES, 2 h ES, 4 h ES and 6 h ES).

### 11. Statistic Analysis

All quantitative data are presented as mean±SD. To compare the statistic difference between any pair of data *t*-test was used to calculate the *p* value that is considered significant when it is less than or equal to 0.05.

## Results

### 1. ES Produced No Side Effect on the Human Skin Fibroblasts

**Figure 1:**
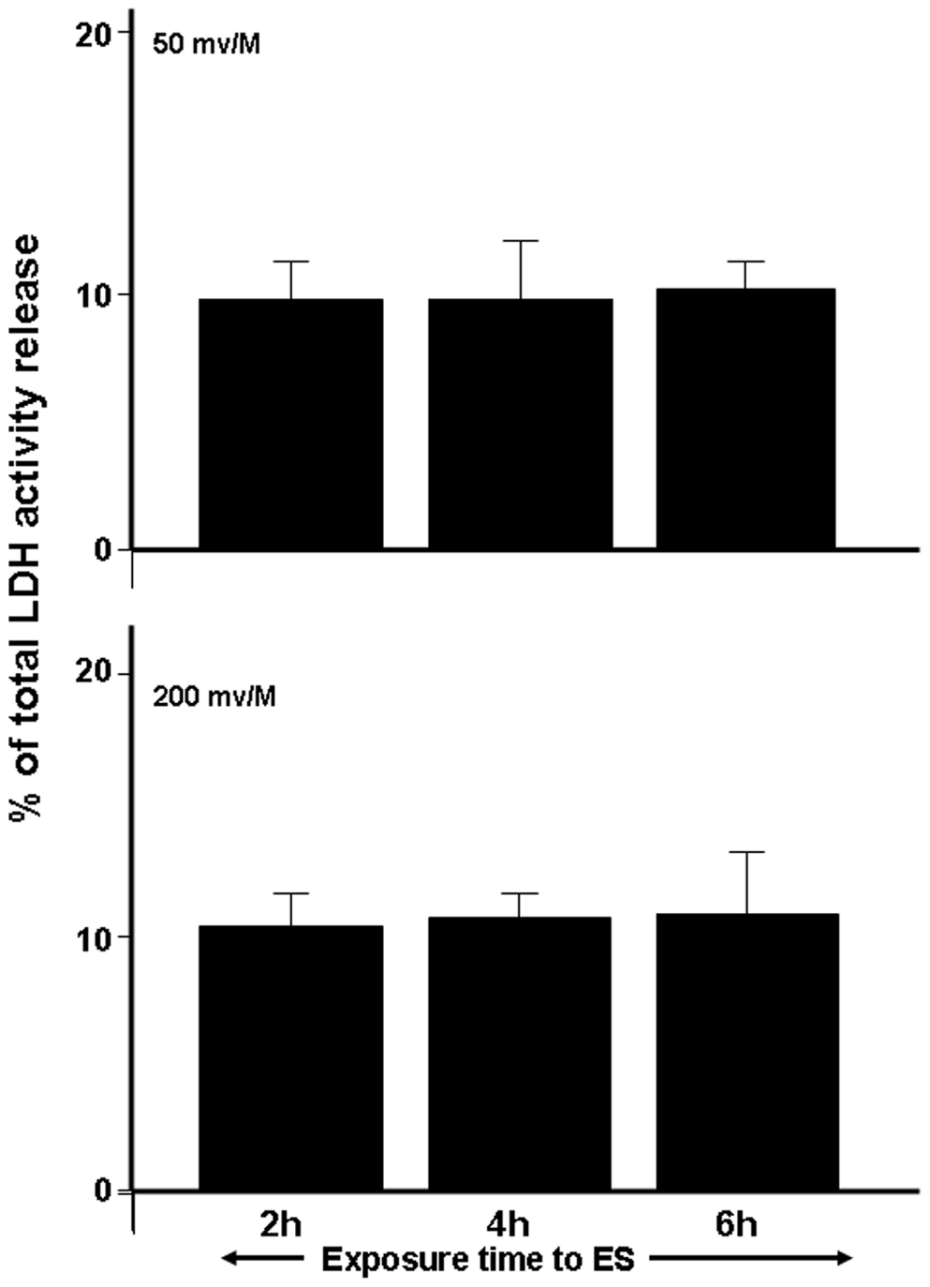
Electrical stimulation (ES) had no cytotoxic effect on human skin fibroblasts. Cells were cultured and subjected to ES at 50 or 200 mV/mm for various periods. Culture supernatants were collected and used to measure LDH activity release, as described in the M & M section. Data are means ± SD, *n*  =  5, with p<0.05 indicating the significant difference versus the control (non-exposed cells).

**Figure 2:**
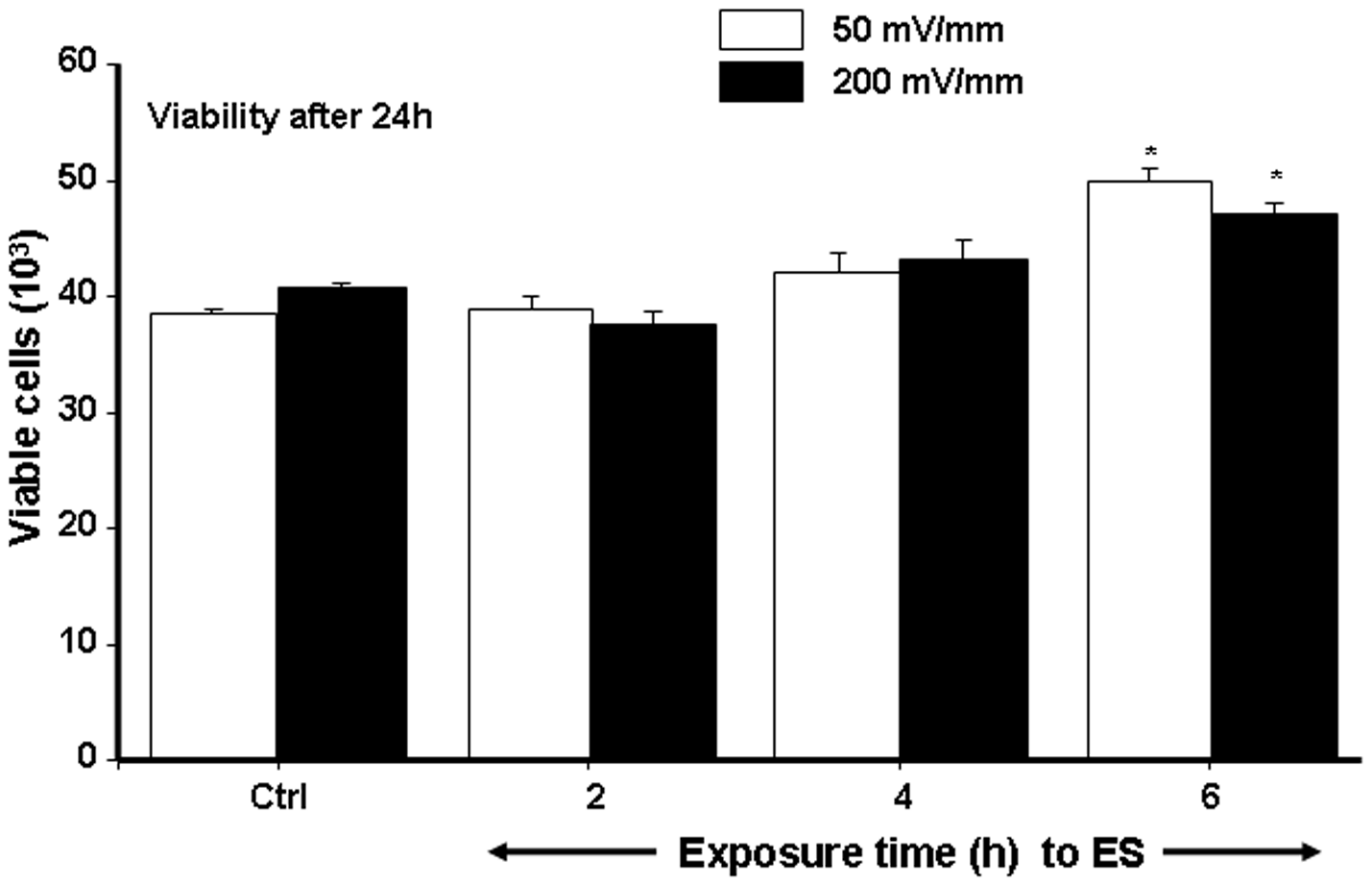
Effect of ES on dermal fibroblast viability. Cells were seeded on the conductive PPy/HE/PLLA membranes and cultured for 24 h, then exposed to ES of 50 or 200 mV/mm for 2, 4, or 6 h, and subsequently cultured for an additional 24 h. The cells were then detached from each membrane and cell viability was ascertained by trypan blue exclusion assay (*n* = 5). The ES-exposed and non-exposed cultures were compared, with the difference considered significant at p<0.05.

To investigate the effect of ES on cell membrane integrity, LDH activity in the culture medium was measured. The release of LDH activity showed no significant difference when human skin fibroblasts were exposed to 50 or 200 mV/mm for 2, 4, or 6 h and cultured for 24 h post-exposure compared to non-ES-exposed cells (Ctrl) (**Figure 1**). It is also interesting to note that no difference was found in LDH levels between the ES intensities and exposure times, which thus suggests that ES had no toxic effect on the fibroblasts. These data are supported by the cell viability analysis. As shown in **Figure 2** **,** no difference in cell density was observed between the ES-exposed and non-exposed fibroblast cultures. There was also no difference when cells were exposed for 2 or 4 h to either 50 or 200 mV/mm. It is, however, interesting to note that the exposure to ES for 6 h produced a significantly (p<0.001) higher cell count compared to the non-exposed control culture. These results clearly demonstrate the absence of any toxic effect of ES on human skin fibroblasts.

### 2. ES Promoted Cell Migration and Wound Closure

**Figure 3:**
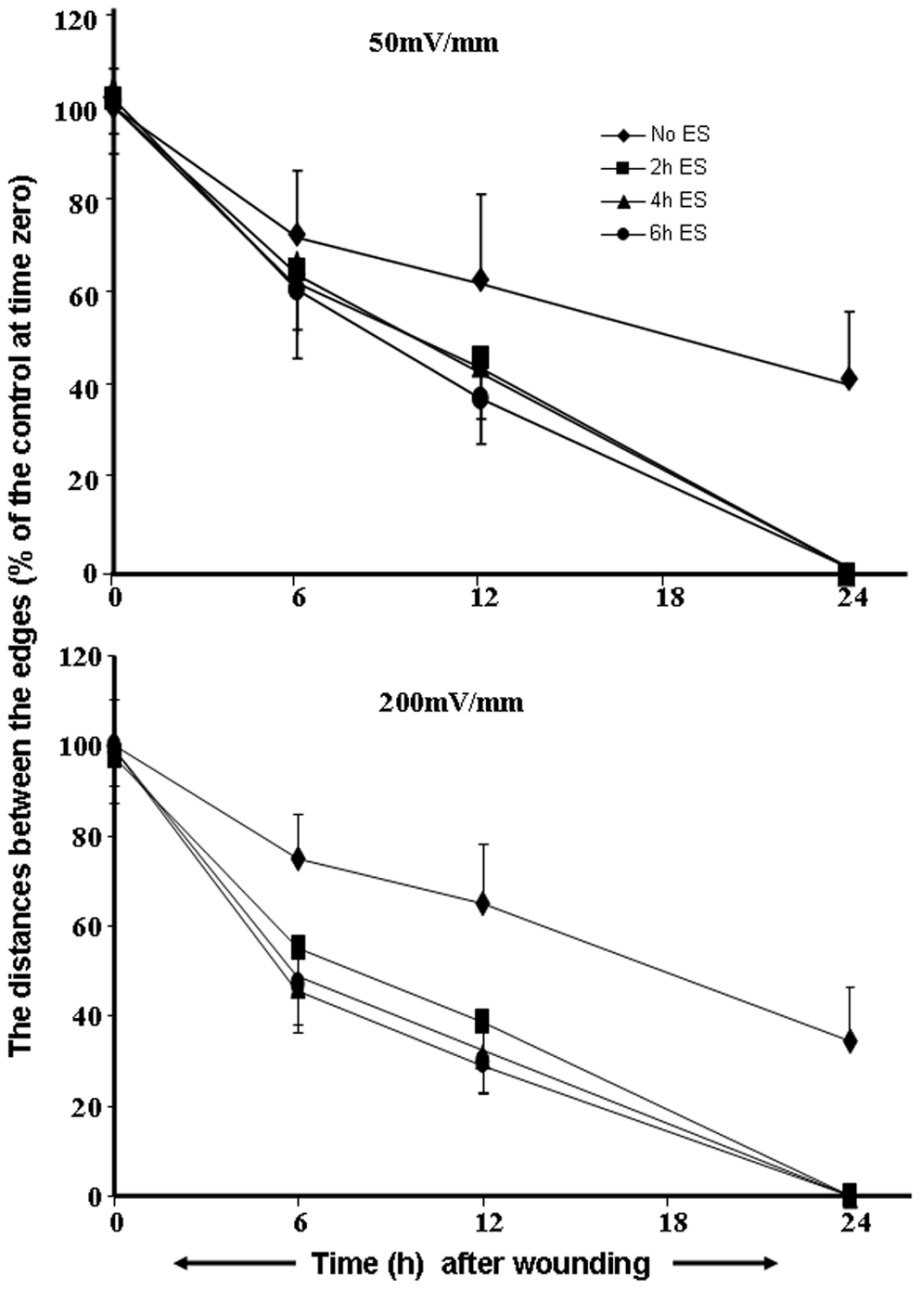
Effect of ES on dermal fibroblast migration/repair. Cells were cultured on PPy/HE/PLLA conductive membranes for 24 h, then exposed to ES for 2, 4, or 6 h, followed by a culture without ES for 24 h. The cells were detached from each membrane, seeded in Petri dishes, and cultured up to 100% confluence. Scratches were then made on each monolayer and the medium was refreshed, with the cultures maintained for various time periods prior to observation and determination of the wound recovery. Values are means ± SD (*n* = 5). The ES-exposed and non-exposed cultures were compared, with the difference considered significant at p<0.05.

Because ES was found to be non-toxic, we investigated whether or not it promoted fibroblast growth and migration. This was determined by means of a scratch wound closure experiment. Wounds were created in skin fibroblast monolayers, and cell migration from the edge of the scratch toward the center, namely wound closure, was recorded at 6, 12, and 24 h post-wounding. The ES-exposed fibroblasts actively migrated from both edges and had closed the entire wound at 24 h (**Figure S1**). This healing rate was much more rapid than that of the non-exposed cells which left 30–40% of the wound unhealed at 24 h (**Figure 3**). Further analysis showed that compared to the non-exposed controls, a significant (p<0.01) reduction of wound distance was observed at 12 h and 6 h for the cells treated with 50 and 200 mV/mm, respectively, with a higher migration rate observed for the cells treated at 200 mV/mm (**Figure 3**). Interestingly, the migration rate of all of the cultures was almost linear, thus it was possible through extrapolation to predict that the complete healing of the non-exposed cultures would be much slower than that of the ES-exposed cells. Cell migration was comparable with 2, 4, or 6 h exposure to ES. This increased cell migration may have involved growth factors such as fibroblast growth factors.

### 3. ES Promoted FGF-1 and FGF-2 Release by the Skin Fibroblasts

**Figure 4:**
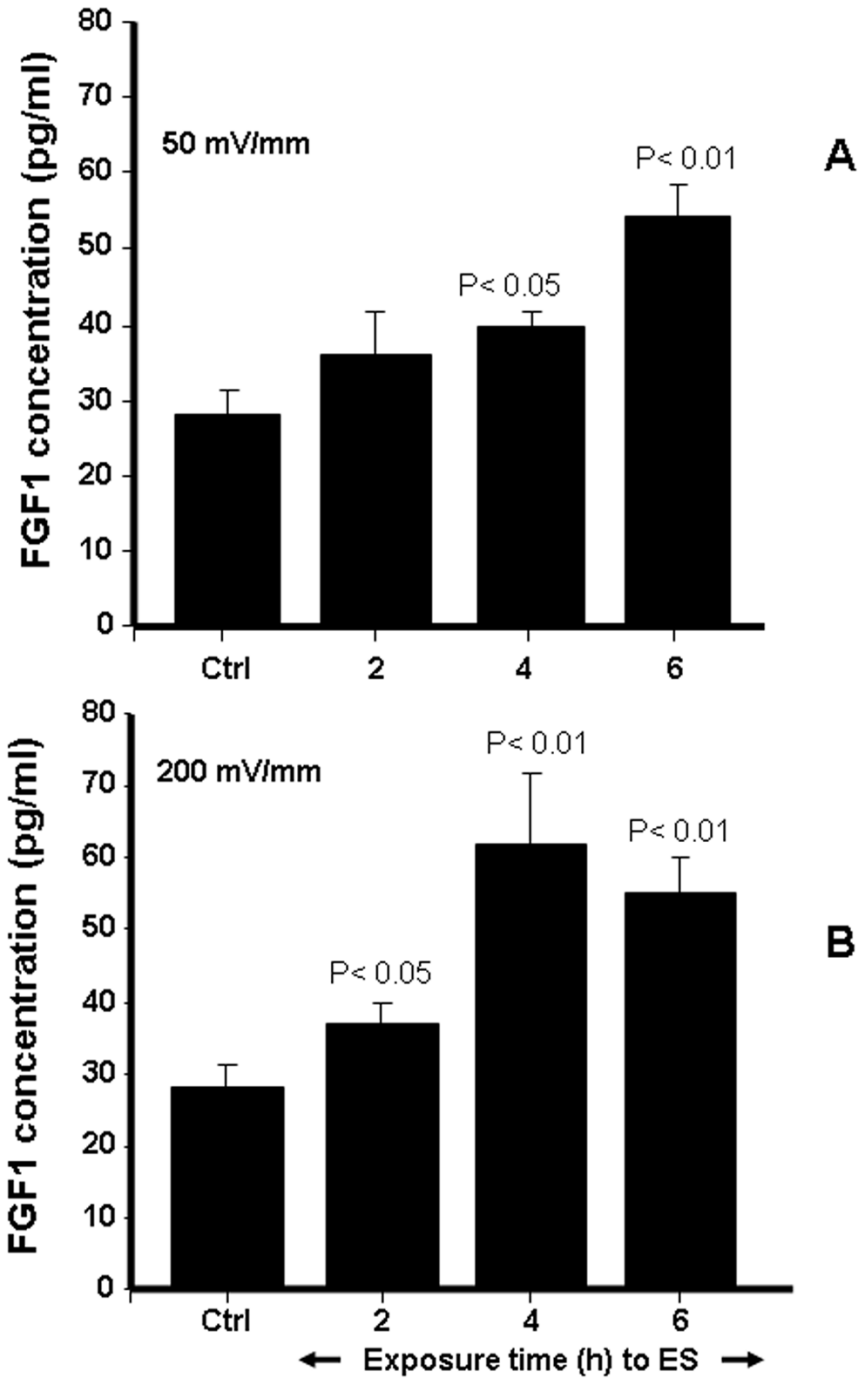
Effect of ES on FGF-1 secretion by the dermal fibroblasts. Cells were cultured on PPy/HE/PLLA conductive membranes for 24 h, followed by exposure to ES for 2, 4, or 6 h. A medium containing only 1% FBS-supplemented DME was used to culture the cells for 24 h post-exposure to ES. The supernatants were then collected and FGF-1 concentrations in all conditions were quantified by sandwich enzyme-linked immunosorbent assays. Values are means ± SD (*n* = 5). The ES-exposed and non-exposed cultures were compared, with the difference considered significant at p<0.05.

**Figure 5:**
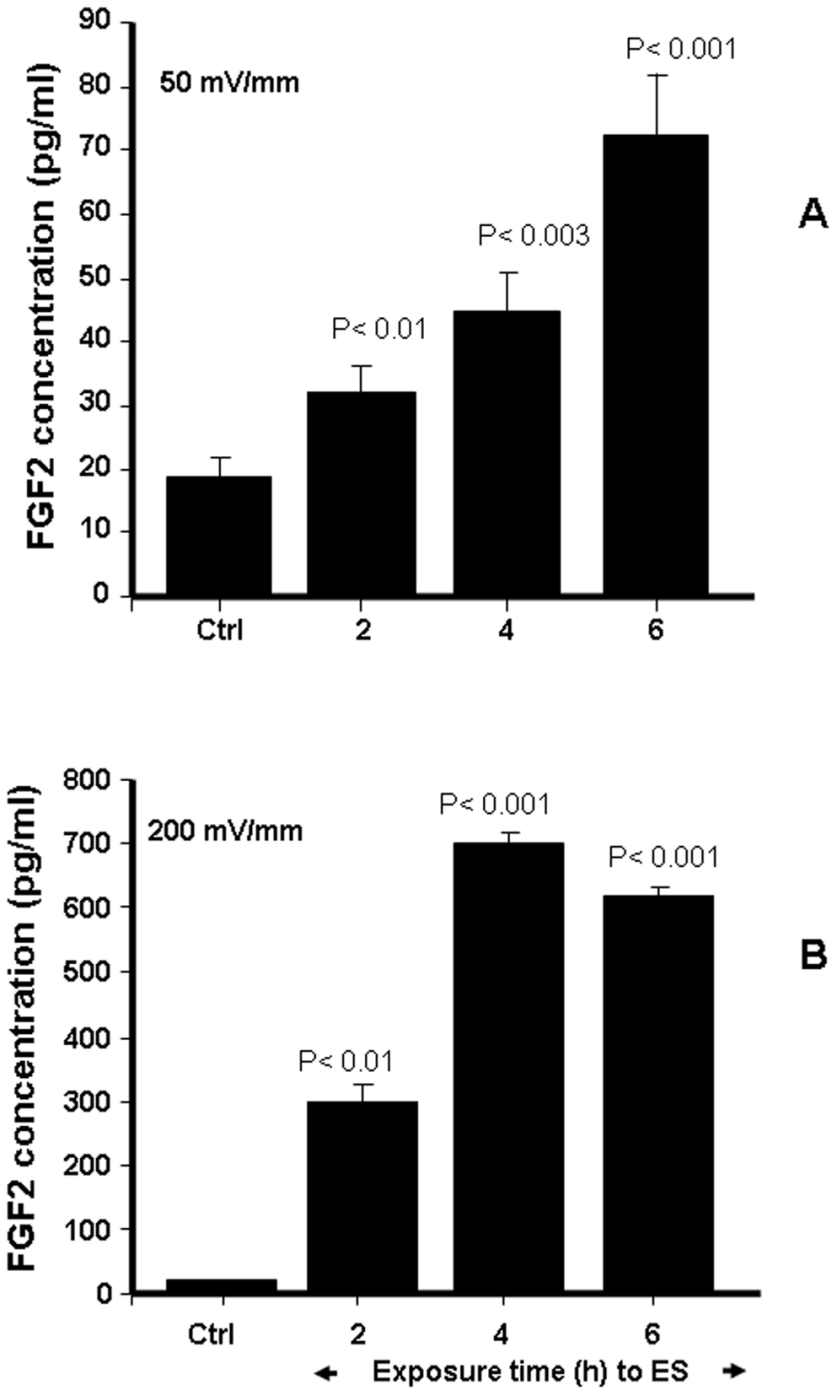
Effect of ES on FGF-2 secretion by the dermal fibroblasts. Cells were cultured on PPy/HE/PLLA conductive membranes for 24 h followed by exposure to ES for 2, 4, or 6 h. A medium containing only 1% FBS-supplemented DME was used to culture the cells for 24 h post-exposure to ES. The supernatants were then collected and FGF-2 concentrations in all conditions were quantified by sandwich enzyme-linked immunosorbent assays. Values are means ± SD (*n* = 5). The ES-exposed and non-exposed cultures were compared, with the difference considered significant at p<0.05.

Without ES, the amount of FGF-1 produced by the fibroblasts was approximately 30 pg/ml (**Figure 4**). Following exposure to 50 mV/mm ES, this significantly (p<0.01) increased to almost twice the basal level. This increase was induced at as short as 2 h of exposure, apparently proportional to stimulation time and attained its maximum at 6 h exposure (**Figure 4A**). ES at 200 mV/mm also promoted FGF-1 secretion. FGF-1 levels increased from 30 pg/ml for the control to 40, 69, and 72 pg/ml for the cells exposed to 2, 4, and 6 h of ES, respectively (**Figure 4B**). At 200 mV/mm, the maximum level of FGF-1 production was reached following 4 h of exposure to ES. FGF-2 secretion was also modulated by ES. As shown in **Figure 5**, basal level FGF-2 concentration was approximately 20 pg/ml (**Figure 5A**); this significantly (p<0.01) increased when the cells were exposed to 50 or 200 mV/mm of ES. At 50 mV/mm, the concentrations were approximately 30, 45, and 70 pg/ml after 2, 4, and 6 h of exposure to ES, respectively. FGF-2 secretion increased significantly more with an exposure to 200 mV/mm than to 50 mV/mm. As early as 2 h of exposure to 200 mV/mm ES, the level of FGF-2 level was over 300 pg/ml, reaching 700 pg/ml after 6 h of exposure. Maximum FGF-2 production appeared after a shorter period of ES in the group exposed to high EF (4 h at 200 mV/mm versus 6 h at 50 mV/mm). Overall data confirm that both 50 and 200 mV/mm upregulated FGF-1 and FGF-2 secretion by the skin fibroblasts.

### 4. ES Promoted Extracellular Matrix Contraction by Increasing α-SMA Expression by the Skin Fibroblasts

**Figure 6:**
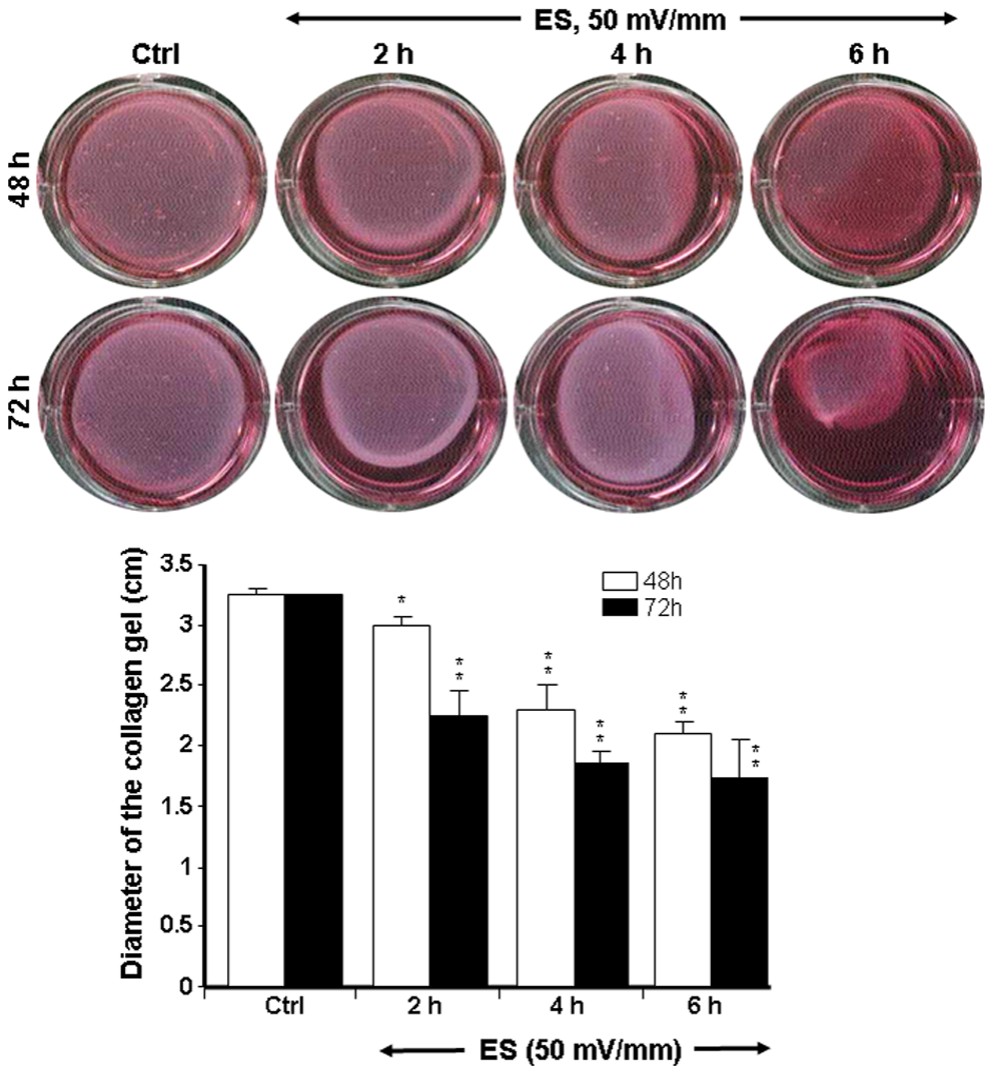
Dermal fibroblasts exposed to 50 mV/mm contracted the collagen gel matrix. Following exposure to ES, the fibroblasts were added to collagen type I gel and cultured. The diameter of each collagen gel was measured at 48 and 72 h. Representative photos show the action of the ES-exposed and non-exposed cells on collagen gel contraction. The diameters of the collagen gels are presented. Values are means ± SD (*n* = 5). The ES-exposed and non-exposed cultures were compared, with the difference considered significant at p<0.05.

Collagen gel assay is a convenient method to investigate cell behavior and three-dimensional (3D) matrix remodeling that more closely resembles cell behavior *in vivo* [36]. To establish whether ES was beneficial to tissue repair by boosting the contractile capacity of fibroblasts, we investigated the collagen gel contraction by ES-exposed fibroblasts. As shown in **Figure 6**, the diameter of the collagen gel containing the non-exposed fibroblasts (Ctrl) remained relatively unchanged following culture for 48 and 72 h. However, for the gel seeded with fibroblasts exposed to 50 mV/mm ES, the diameter of the collagen gel went from close to 3.3 cm in the control group down to approximately 2.2 cm in the group exposed to ES for 6 h.

**Figure 7:**
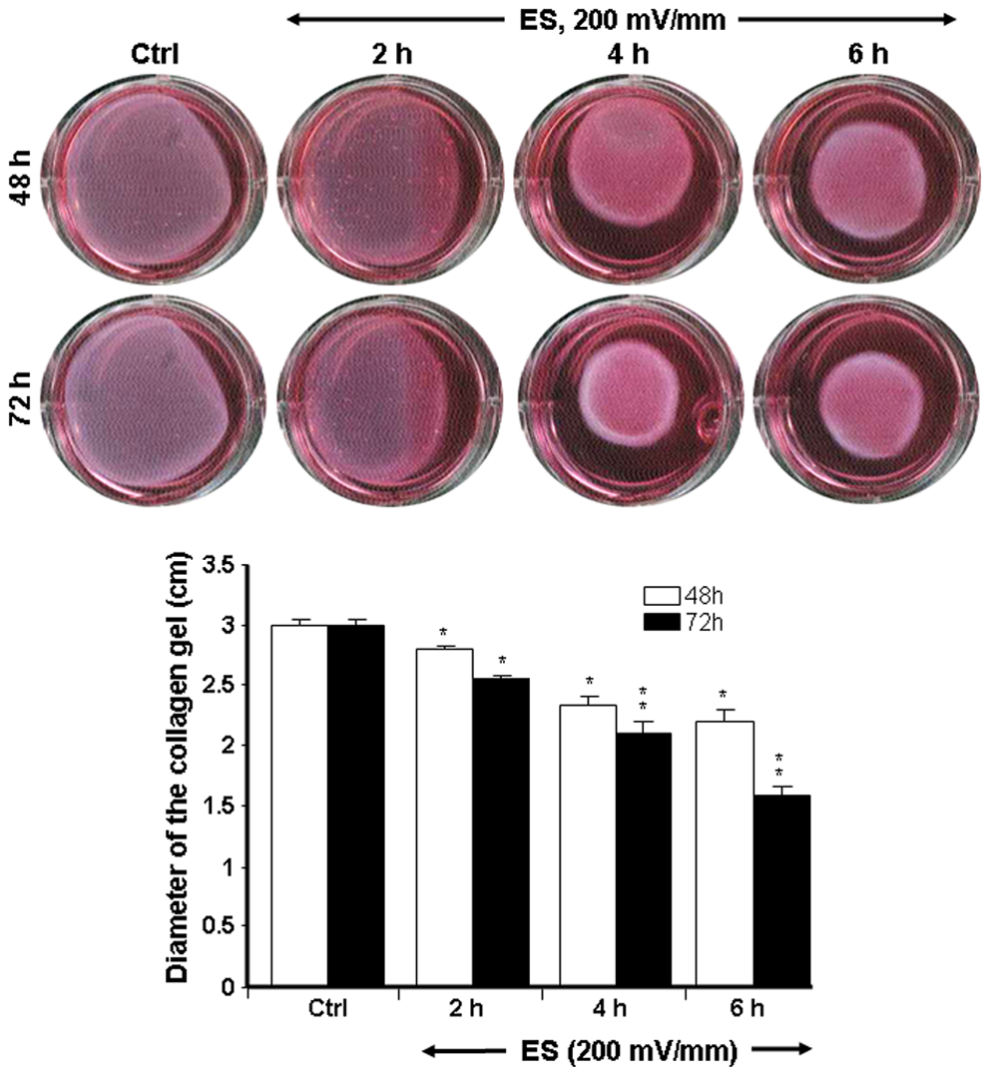
Dermal fibroblasts exposed to 200 mV/mm highly contracted the collagen gel matrix. Following exposure to ES, the fibroblasts were added to a collagen type I gel and cultured. The diameter of each collagen gel was measured at 48 and 72 h. Representative photos show the action of the ES-exposed and non-exposed cells on collagen gel contraction. The diameters of the collagen gels are presented. Values are means ± SD (*n* = 5). The ES-exposed and non-exposed cultures were compared, with the difference considered significant at p<0.05.

The highest collagen gel contraction was recorded at 72 h for the group exposed for the longest period to ES (**Figure 6**). Comparable results were obtained with 200 mV/mm. **Figure 7** shows that the size of the collagen gel in the control was approximately 3.0 cm. This significantly (p<0.01) decreased in the gels containing fibroblasts exposed to 200 mV/mm for 2, 4, and 6 h. It is important to note that the longer the ES exposure time, the higher the level of contraction of the collagen gel. Similarly, the reduction of gel size was also greater at a stronger EF.

**Figure 8:**
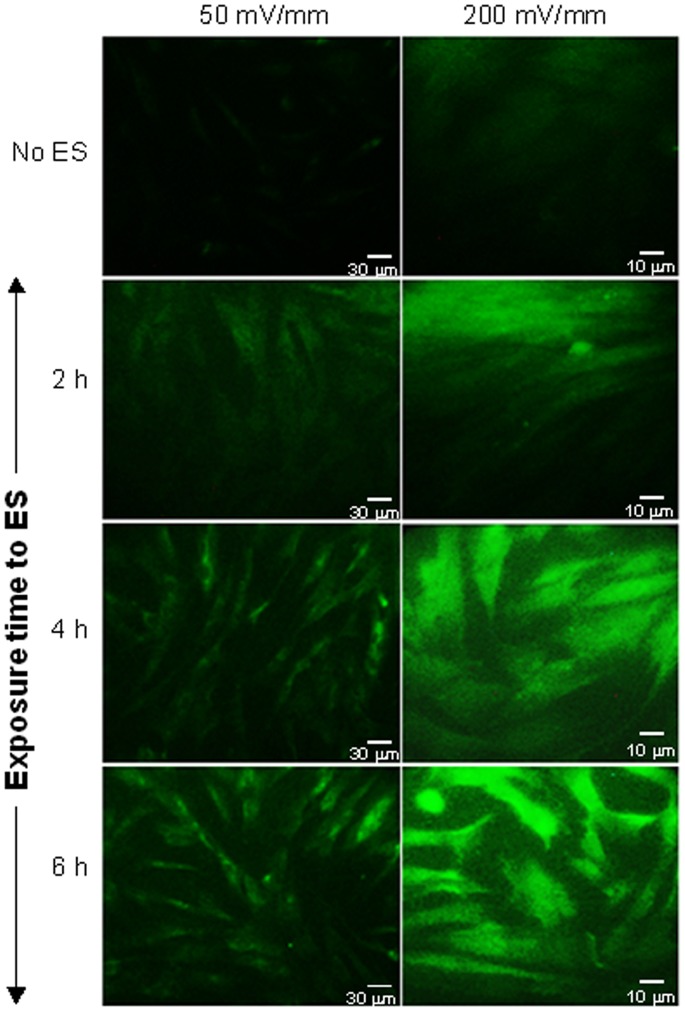
Effect of ES on fibroblast α-SMA expression. Dermal fibroblasts were seeded on conductive PPy/HE/PLLA membranes followed by exposure to 50 or 200 mV/mm for various periods. The cells were then detached from the conductive membranes, washed, seeded on coverslips, and cultured up to 70% confluence. The cells were then stained using relevant monoclonal antibodies. Cytoplasmically immunolabelled myofibroblasts are presented.

Overall data demonstrate that ES augmented fibroblast contractile activity, which may be due to the expression of contractile fibers, such as α-SMA fibers. Immunofluorescence staining confirmed that the non-exposed cells expressed no α-SMA contractile fibers. However, following 2 h of exposure to ES, there was evidence of cells displaying a high staining to α-SMA (**Figure 8**). The stain intensity increased proportionally to exposure time and ES intensity**.** These data support those related to the extracellular matrix contraction.

### 5. The Effect of ES on Fibroblast Growth was Maintained Over the Cell Passages

**Figure 9:**
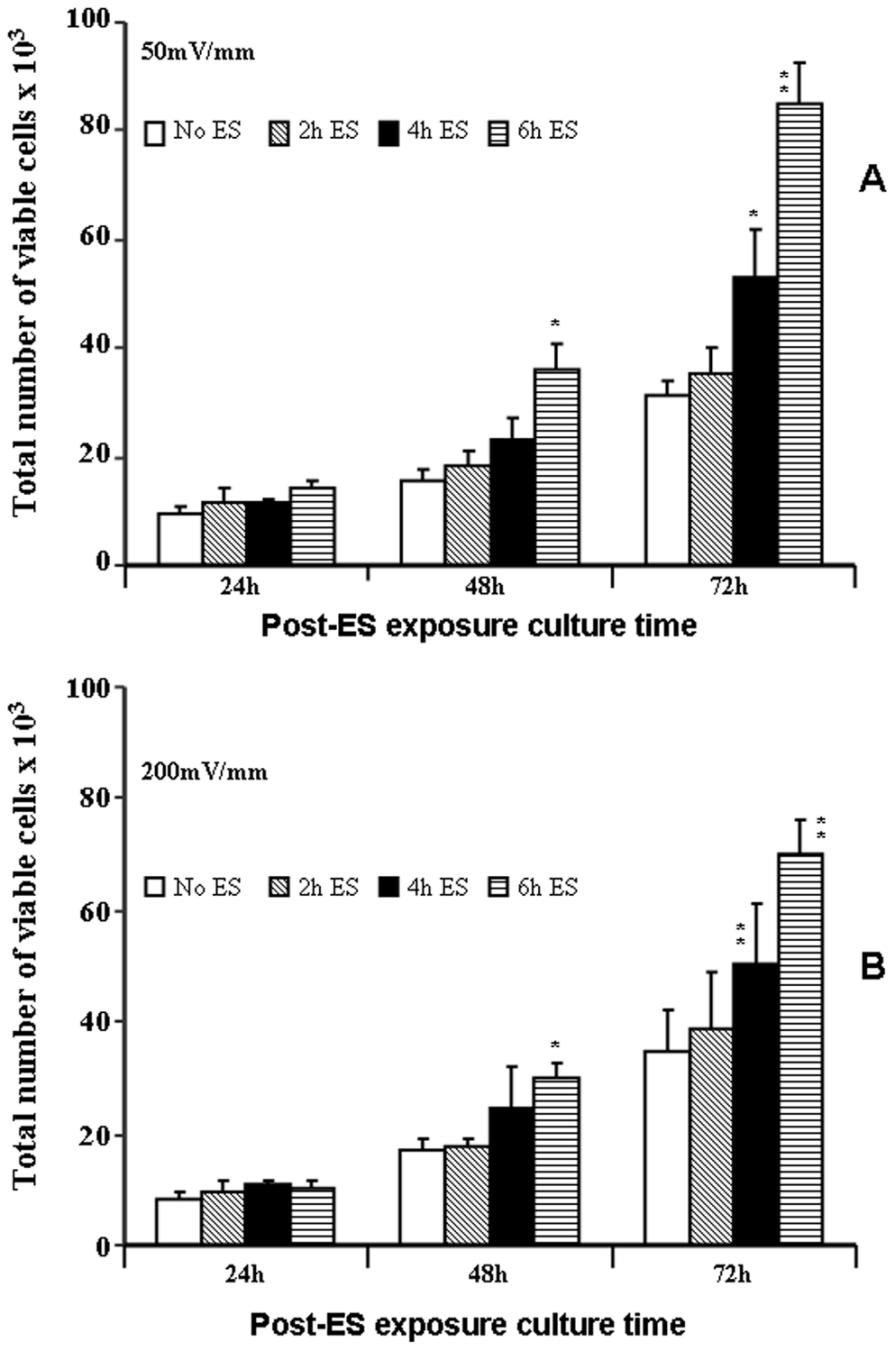
The proliferative capacity of fibroblasts was maintained and elevated following exposure to ES for 2, 4, or 6 h. The cells were then detached and used to investigate their proliferative capacity at longer periods (24, 48, and 72 h) post-exposure to ES. Viability was determined by trypan blue exclusion assay. Values are means ± SD, (*n* = 6). The ES-exposed and non-exposed cultures were compared, with the difference considered significant at p<0.05.

As cell proliferation, growth factor production and cell contractile capacity were promoted by ES, we hypothesized that ES could promote cell growth and that this stimulatory effect could be transmitted from one cell generation to another. To address this possibility, fibroblasts were exposed to ES and subsequently cultured for 24 h. They were then trypsinised and subcultured for 24, 48, and 72 h to measure cell growth. As shown in **Figure 9A**, the subculture of the cells exposed to 50 mV/mm ES for 4 and 6 h recorded a faster growth rate compared to that of the controls, and that this was evident at both 48 and 72 h. The most significant (p<0.01) growth was recorded by the fibroblasts exposed to 50 mV/mm for 6 h, and similar results were obtained at 200 mV/mm. **Figure 9B** illustrates that while the difference was not significant after 24 h of subculture, fibroblast growth after 48 and 72 h of subculture was indeed greater (p<0.01) for the cells exposed to ES for 4 and 6 h. This was confirmed by the rate of cell growth. While cells grew exponentially in all cultures, the growth rate of the cells stimulated for 4 h and 6 h was higher. Indeed, the ratios of viable cells of 48 h and 24 h (48 h/24 h) increased from 1.6 for both no-ES and 2 h-ES to 2.0 and 2.6 for the cells stimulated for 4 (4 h ES) and 6 hours (6 h ES), respectively. The trend of increase in cell growth rate with ES was similar at both ES strengths (**Figure S2**). These observations demonstrate that the stimulatory effect of ES on fibroblast growth was maintained from one passage to another and therefore promoted wound healing.

## Discussion

This study clearly demonstrates that ES had no cytotoxic effect on human skin fibroblasts seeded on PPy/HE/PLLA conductive membranes. The fibroblasts adhered well and proliferated exponentially following various exposure periods to ES. Although cells were found to adhere to both the ES-exposed and non-exposed conductive membranes, the ES-exposed membranes produced a higher number of viable cells (**Figure 2**) and comparable LDH activity (**Figure 1**), compared to the non-exposed membranes. Of particular interest is that cell viability/density increased with longer exposure periods (4 and 6 h, **Figures 2** **and** **9**). Thus the relevant cell fate processes to be considered in this context included cell adhesion, growth, and proliferation. These processes may depend on growth mediator expression within the cell, and are enhanced following exposure to EF [37]. One study reported that cell proliferation and spreading on the surface of biomaterials were enhanced within a narrow window of voltage/frequency of ES [38]. This led us to the hypothesis that ES-exposed fibroblasts may secrete a high level of growth factors such as FGFs, thereby promoting their viability and proliferation. Based on the measurements of the secreted FGF-1 and FGF-2, we demonstrated that ES through the PPy/HE/PLLA conductive membranes significantly (p<0.01) increased FGF secretion. It was therefore deemed reasonable to further hypothesise that the enhanced FGF-1/FGF-2 secretion by ES was due to the effect of EF on the internal functions of the cell. ES was shown to enhance cell proliferation by increasing the cyclic AMP content (cAMP) and by stimulating gap-junction communication [39]. It has been previously reported that ES led to elevation of FGF-2 in the cytoplasm of neurons and in the nucleus of reactive astrocytes [40]. Also, Düsterhöft et al. reported that ES modulates the expression level of FGF-1, FGF-2 and their receptors (FGFR1, FGFR4) in the tibialis anterior muscle of hypothyroid rat, as well as in satellite cell cultures derived from normal rat [41]. This is supported by our observations demonstrating that ES promoted FGF-1 and FGF-2 secretion by human skin fibroblasts. Part of a large family of polypeptides, FGFs are considered potent regulators of cell growth and differentiation [42], [43]. The most predominant FGFs, FGF-1 and FGF-2, are involved in fibroblast growth and trigger angiogenesis, cell migration, and wound healing [44]. Thus their up-regulation by ES is likely to promote skin fibroblast migration. To test this hypothesis, ES-exposed skin fibroblasts were subjected to an *in vitro* wound scratch assay, which showed that ES at 50 and 200 mV/mm elevated fibroblast migration and wound closure. It was reported that under EFs ranging from 200–400 mV/cm, fibroblasts from different species migrated toward the cathode, although corneal stroma fibroblasts and pulmonary artery fibroblasts were shown to migrate toward the anode [19], [20], [45]–[47]. The reason explaining this mixed polarity among different cell types remains unclear. The use of electrodes in these studies also complicated the interpretation of the results because of the potential disturbance of the electrodes to the culture medium. Such disturbance includes the electrophoresis of charged species in culture medium and the electrolysis of water if there exits a direct contact of metal electrode and culture medium and a high enough electrical potential. In our wound healing study, in the absence of ES, fibroblasts migrated from both edges of the wound to cover the cell-free surface, demonstrating an elevated migration rate with no polarity. This study therefore provides solid evidence to show that the elevated cell migration and viability were due to the change in cell functions and structures induced by ES. Both EF strength and exposure time are important factors in regulating cellular processes. Endogenous EFs vary depending on tissue composition and location. For example, an EF of approximately 150 mV/mm is typical in the epidermis and specifically in the cornified layer of the epidermis [48]. On the other hand, the EF in fibroblast-populated connective tissue (such as the dermis) is weak because the electrical resistance in this tissue is relatively low, thus preventing the establishment of sufficiently high EF. The strongest endogenous EFs and currents were measured at the wound edge [49], [50], which were helpful to heal the wound. Human skin fibroblasts failed to respond within 1 h to an EF of 100 mV/mm [51]. However, Guo et al. (2010) [19] showed that at a longer exposure time, fibroblasts migrated directionally to the anode. These authors also reported that fibroblasts migrated at lower rates under an EF of 50 mV/mm than under 100 mV/mm. These findings are supported by our results showing that growth factor secretion and fibroblast migration were greater at 200 mV/mm than at 50 mV/mm. Exposure time to ES is also important for cell activation [19]. In our study, the fibroblasts exposed to ES for longer periods (4 and 6 h) secreted higher concentrations of FGF-1 and FGF-2 and migrated faster than did the fibroblasts exposed for 2 h and the non-exposed cells. Cell migration toward the wound is a crucial step in all wound healing processes in mammals. This response precedes and positions cells, such as fibroblasts, at the appropriate areas for proliferation and differentiation. Studies have proposed that endogenous EFs at wounds serve as a modulator to help cells migrate during wound healing [52], [53]. Other studies in the last decade have provided convincing evidence on the role of EFs in wound healing [54], [55]. Important molecules such as EGF receptors, integrins, V-ATPase H+ pump, and PI3 kinase/Pten [56]–[59] were shown to be involved in EF-induced cellular reactions. Because of the participation of these signaling molecules, we hypothesised that ES-exposed fibroblasts may be involved in wound closure through their activation/differentiation into myofibroblasts. These cells, key players in the wound healing process, are responsible for cell-mediated matrix contraction [60], [61]. Through a contraction of their actin cytoskeleton, myofibroblasts at the wound site are able to reduce the initial size of the wound, thereby contributing to tissue repair. In light of this, our study demonstrated for the first time that ES-exposed skin fibroblasts were able to reduce the size of a 3D collagen matrix. These findings concur with those of an animal study showing that direct current EF favorably affected collagen synthesis and wound closure/contraction [62]. Wound contraction is predominantly myofibroblast-dependent [63]. These cells express the morphological and functional characteristics of smooth muscle through the expression of α-SMA, which is essential to wound contraction [64], [65]. The ES-enhanced fibroblast migration and extracellular matrix contraction observed in our study can be explained by the high expression of α-actin protein by the skin fibroblasts following exposure to ES at 50 and 200 mV/mm. It is important to note the potential dose effect of ES, as the highest level of α-actin expression was obtained at the highest ES intensity (200 mV/mm) and for the longest exposure period (6 h exposure to ES). α-SMA modulation by ES was previously reported in vascular smooth muscle cells (VSMC). Indeed, Rowlands and Cooper-White in 2008 reported differences in the expression of contractile phenotype markers (α-SMA and smooth muscle myosin heavy chain) between the non-stimulated and 5 Hz stimulated VSMCs, suggesting that ES through conductive material can direct the phenotype of VSMCs [66]. These are supportive to our data about ES up-regulated expression of α-SMA in skin fibroblasts. This suggests one of a number of ways that ES helps wound healing by increasing the number of fibroblasts expressing α-SMA in the wound, which in turn benefits wound healing. The effects of ES on skin fibroblasts may be either transient or maintained over a long period of time. Using fibroblasts first exposed to ES, then removed and subcultured for 24, 48, and 72 h, we demonstrated for the first time that during these subculture times, high levels of cell growth were maintained. Maintaining this growth rate for such a long time is physiologically relevant, as cells are continuously involved in the various phases of the wound healing process [67]. This finding is also potentially significant for clinical application, as the transplantation of ES-exposed autologous cells is much easier than is in situ ES. While ES using electrodes has been reported to accelerate axon regeneration and muscle reinnervation in clinic setting [68], ES to open wound can be technically challenging, such as how to design electrodes to provide uniform stimulation to the wounds of various size and forms, and the risk of infection while applying electrodes to the wound. On the other hand, skin fibroblasts can be easily harvested from the patient and electrically stimulated under sterile condition. The transplantation of such electrically stimulated autologous fibroblasts either as cell suspension or in collagen gel as skin equivalent is straightforward and can be performed repeatedly if required. However detailed studies are necessary to investigate the behaviors of the stimulated fibroblasts in real wound, such as the interactions of these stimulated cells with other cell types such as keratinocytes.

### Conclusions

This study demonstrated that constant ES at 50 and 200 mV/mm mediated by conductive polymer substrate promoted skin fibroblast growth, enhanced cell migration, increased FGF-1 and FGF-2 production and induced fibroblasts to myofibroblasts transdifferentiation. It is also demonstrated that such modified cellular behaviors were inherited by the cells up to 3 days in culture. Our overall findings thus suggest the beneficial effect of ES on wound healing processes.

## Supporting Information

Figure S1 **Migration of fibroblasts following exposure to ES for 2, 4, or 6 h.** The cells were then detached from the conductive membrane, seeded in Petri dishes, and cultured up to 100% confluence. Scratches were then made on each monolayer and the culture medium was refreshed. The cultures were maintained under the appropriate conditions, observed, and photographed at various time points. Scale bars: 50 µm.
(TIF)

Figure S2 **Growth rate of the sub-cultures of the ES-exposed fibroblasts.** Following cell culture and viability evaluation, the rate of cell growth at 48 h and 72 h was calculated by dividing the viable cell numbers at 48 h and 72 h with those at 24 h and 48 h, respectively.
(TIF)
