# Supplementary figures and images for: Electrical Stimulation Promotes Wound Healing by Enhancing Dermal Fibroblast Activity and Promoting Myofibroblast Transdifferentiation
Source: PLoS One. 2013 Aug 19;8(8):e71660. doi: 10.1371/journal.pone.0071660 (PMC3747189; doi:10.1371/journal.pone.0071660)

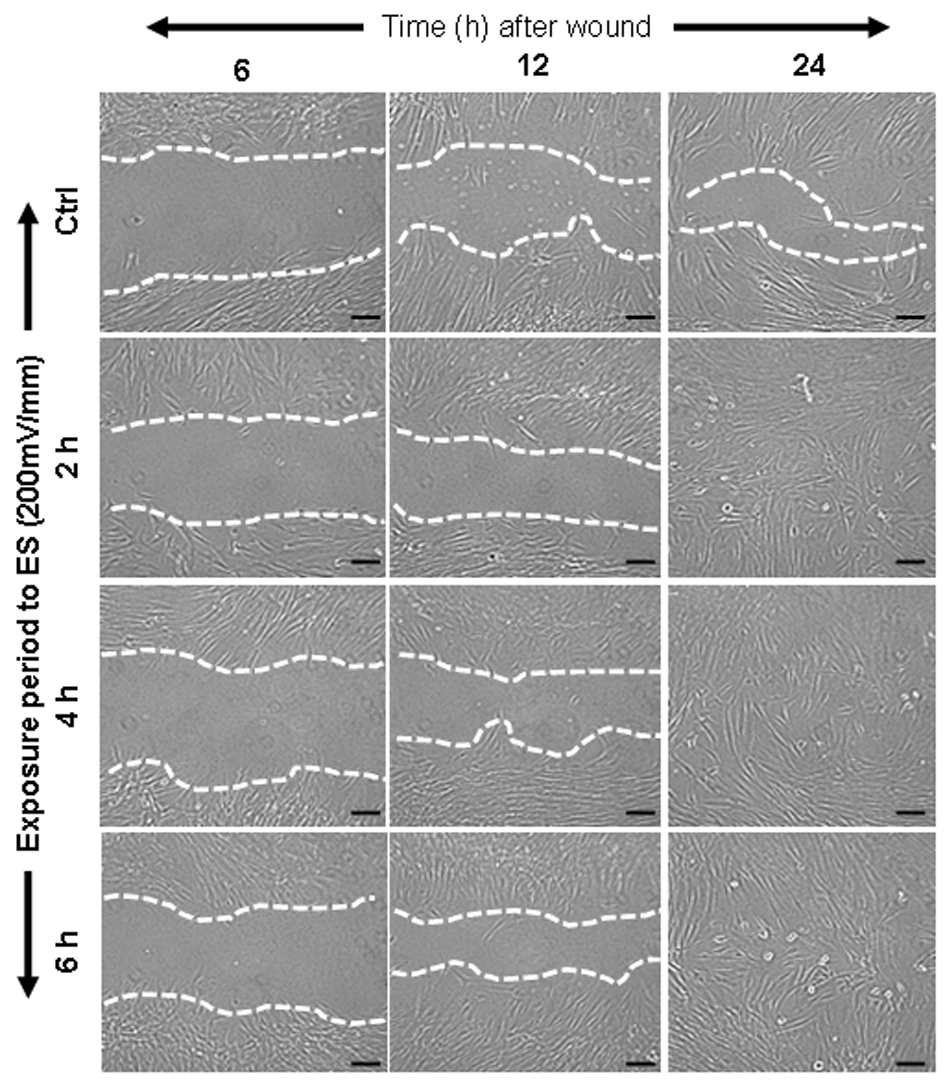

Supplement: Figure S1 — Migration of fibroblasts following exposure to ES for 2, 4, or 6 h. The cells were then detached from the conductive membrane, seeded in Petri dishes, and cultured up to 100% confluence. Scratches were then made on each monolayer and the culture medium was refreshed. The cultures were maintained under the appropriate conditions, observed, and photographed at various time points. Scale bars: 50 µm. (TIF) [file pone.0071660.s001.tif]

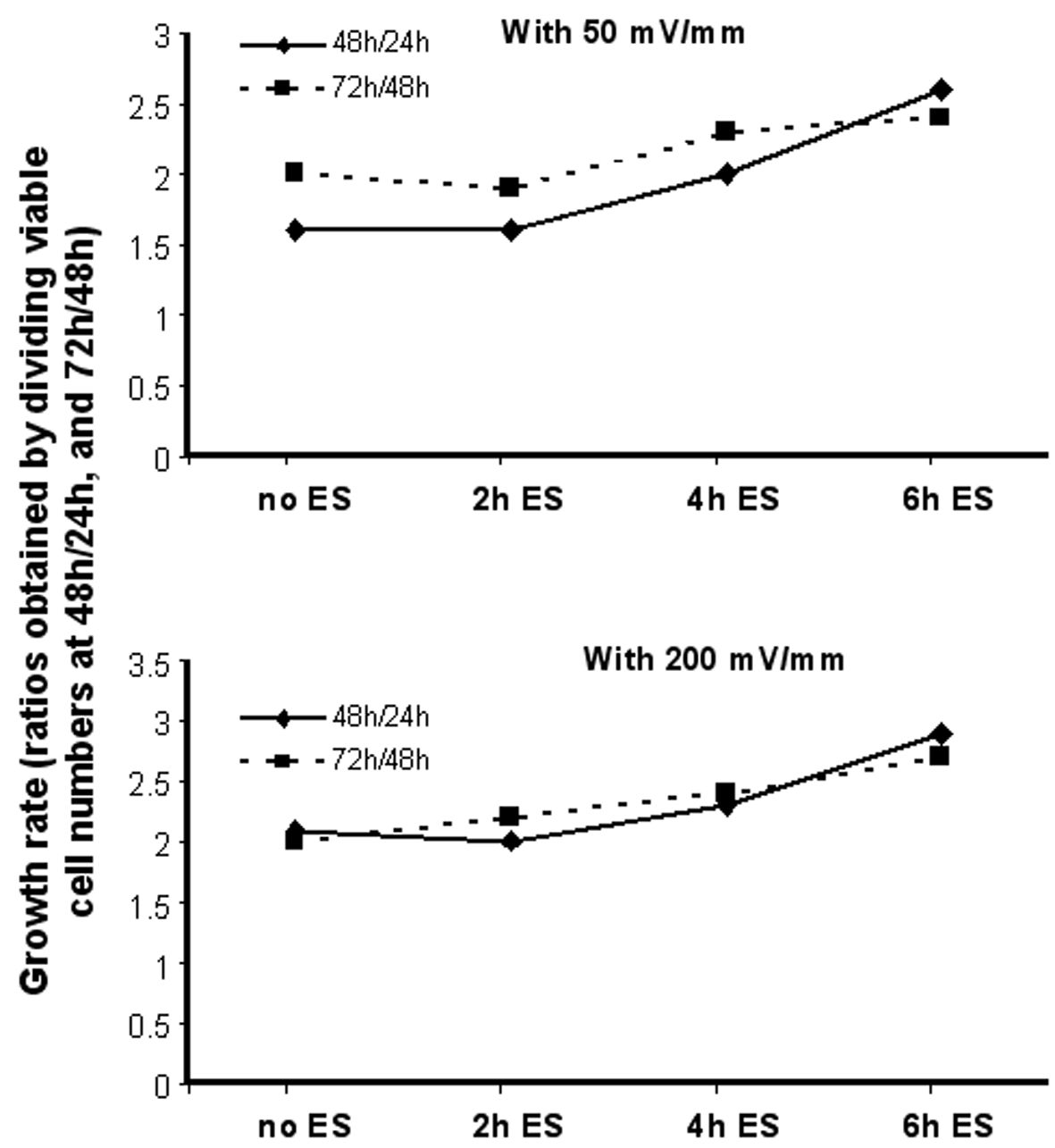

Supplement: Figure S2 — Growth rate of the sub-cultures of the ES-exposed fibroblasts. Following cell culture and viability evaluation, the rate of cell growth at 48 h and 72 h was calculated by dividing the viable cell numbers at 48 h and 72 h with those at 24 h and 48 h, respectively. (TIF) [file pone.0071660.s002.tif]
